# Manganese availability is negatively associated with carbon storage in northern coniferous forest humus layers

**DOI:** 10.1038/s41598-017-15801-y

**Published:** 2017-11-14

**Authors:** Johan Stendahl, Björn Berg, Björn D. Lindahl

**Affiliations:** 1 Department of Soil and Environment, Swedish University of Agricultural Sciences, P.O. Box 7014, SE-75007 Uppsala, Sweden; 2 Department of Forest Sciences, University of Helsinki, P.O. Box 27, FI-00014 Helsinki, Finland

## Abstract

Carbon sequestration below ground depends on organic matter input and decomposition, but regulatory bottlenecks remain unclear. The relative importance of plant production, climate and edaphic factors has to be elucidated to better predict carbon storage in forests. In Swedish forest soil inventory data from across the entire boreal latitudinal range (n = 2378), the concentration of exchangeable manganese was singled out as the strongest predictor (R^2^ = 0.26) of carbon storage in the extensive organic horizon (mor layer), which accounts for one third of the total below ground carbon. In comparison, established ecosystem models applied on the same data have failed to predict carbon stocks (R^2^ < 0.05), and in our study manganese availability overshadowed both litter production and climatic factors. We also identified exchangeable potassium as an additional strong predictor, however strongly correlated with manganese. The negative correlation between manganese and carbon highlights the importance of Mn-peroxidases in oxidative decomposition of recalcitrant organic matter. The results support the idea that the fungus-driven decomposition could be a critical factor regulating humus carbon accumulation in boreal forests, as Mn-peroxidases are specifically produced by basidiomycetes.

## Introduction

Boreal forests have been estimated to store 24% of terrestrial C^1^ and 64% of this C is found below ground^2^. The dynamics of this pool is of major concern, particularly in the light of forthcoming climate change and intensified forestry for timber, fibre, and fuel. However, there is currently no consensus on how below ground C sequestration is regulated across scales and ecosystems and existing models fail to predict stand level C storage^3^. Although high productivity increases input of fresh organic matter, increasing evidence indicates that constrained decomposition rather than high primary production is the most critical factor promoting long-term C sequestration below ground^4,5^. The emerging view on decomposition stresses the importance of both physical and biological interactions, in contrast to the previous one focusing on intrinsic chemical properties of the soil organic matter^6^. For example, feedbacks between plants and soil microorganisms may limit responses to increased production, due to intensified C turnover^7,8^. Critical regulators of below ground C storage recognized in recent studies include mineral protection^9^, nutrient dynamics^10^, rhizosphere priming^11^, and mycorrhizal associations^12^. Thus, climate and productivity alone cannot explain the carbon storage, but the decisive factors are likely to vary between ecosystems. Northern coniferous forests are characterized by low N availability, low soil pH, and a high fungi-to-bacteria ratio, with approximately one third of the total below ground C store found in organic, aerated humus topsoils (mor layers)^13,14^. The average turnover rate of these humus layers is 30–40 years^14^ making the dynamics of this C pool critical in time perspectives relevant for climate change mitigation measures e.g. via changed forest management. Further, the dynamics of the humus layer is critical in regulating ecosystem productivity, since retention of N in humus constrains its availability to plants and limits their production^5^. Still, the regulation of decomposition in these topsoils remains unresolved. Organo-mineral interactions have to play a minor role and instead it has been proposed that soil C is preserved by low pH and the refractory nature of the substrate^15^ with enzymatic depolymerization of macromolecules being a critical regulatory factor.

A sequence of detailed studies of climatic and chemical influences on short-term litter decomposition has identified Mn as an important factor^16–21^. Mass loss has been found to be significantly and positively correlated with Mn concentration in litter of Norway spruce (*Picea abies)* ^22^, common oak (*Quercus robur*)^23^ and common beech (*Fagus sylvatica)* ^24^. In Douglas fir (*Pseudotsuga menziesii*) litter, Keiluweit *et al*.^20^ found that spots with active decomposition were enriched in oxidative Mn^3+^. In particular, Mn appears to be important in stimulating turnover of the most stable litter fraction^16,17,25^, suggesting a central role of Mn in regulating also longer-term C sequestration. However, these previous, incubation-based studies were limited to the initial years of leaf litter decomposition close to the ground surface, whereas the magnitude of the total humus pool depends on longer-term (decades to centuries) processes in the rhizosphere^7^, which are difficult to study directly.

**Figure 1:**
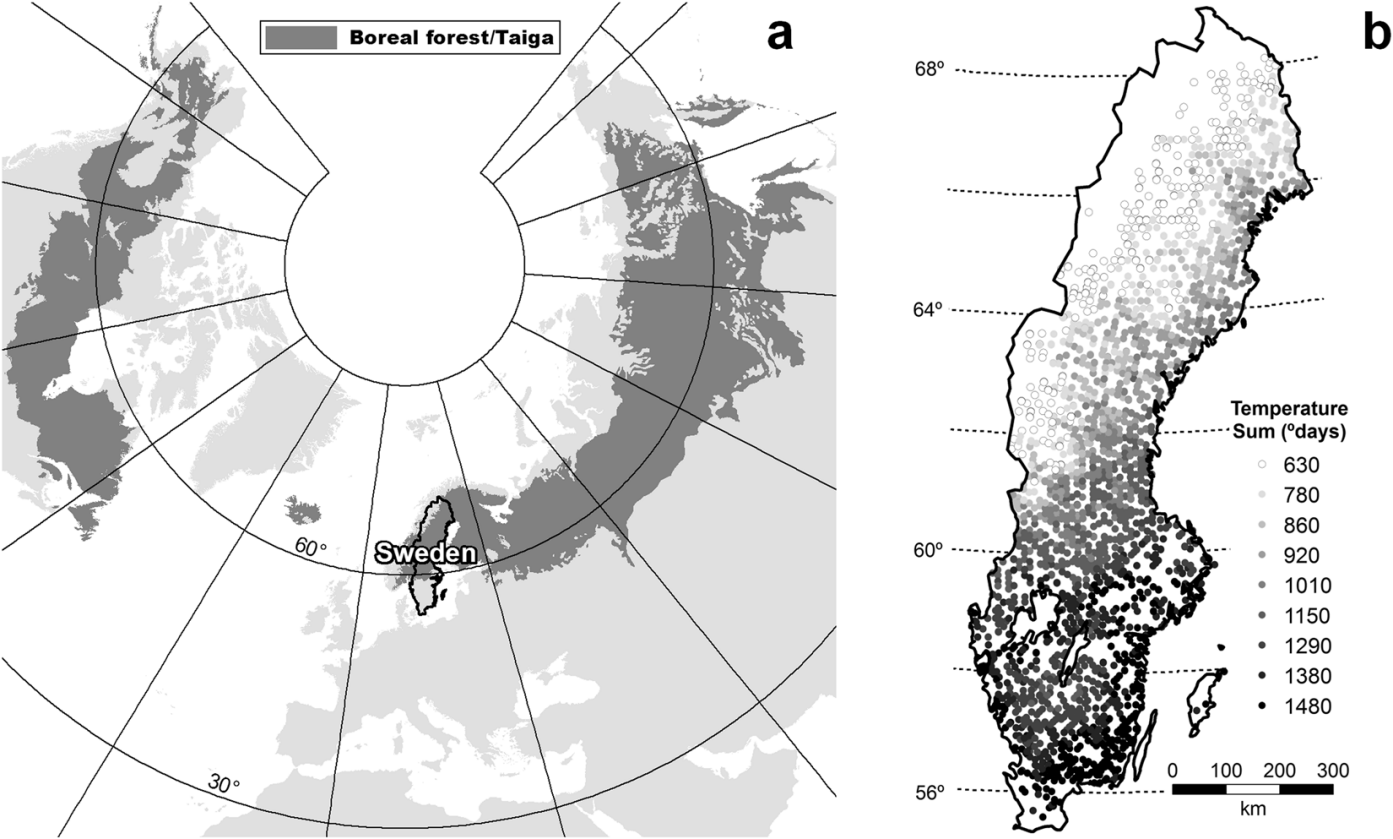
Area of investigation and sampling plot design. Location of Sweden in the Boreal forest/Taiga biome^39^ (map access via https://databasin.org) (**a**) and sites from the Swedish Forest Soil Inventory charted by climate regions (**b**). Maps were produced using ArcGIS 10.2.2 (www.esri.com).

Here we take advantage of data from an extensive inventory of forest soils to examine the importance of Mn for long term below ground C storage at biome level compared to several other potential regulators. Our sampling spans the entire boreal latitudinal range and extends into the temperate region (Fig. 1a). The Swedish Forest Soil Inventory is a sample-based inventory that visits c. 20 000 systematically distributed permanent plots recurrently at 10-year intervals (Fig. 1b). Information on stand, soil, and site characteristics is collected as well as extensive soil chemical data, e.g.; C, N, pH, as well as exchangeable Ca, Mg, K, Na, Mn, and Al (Supplementary Table S1). Data on exchangeable Fe and P were not available for the study.

**Figure 2:**
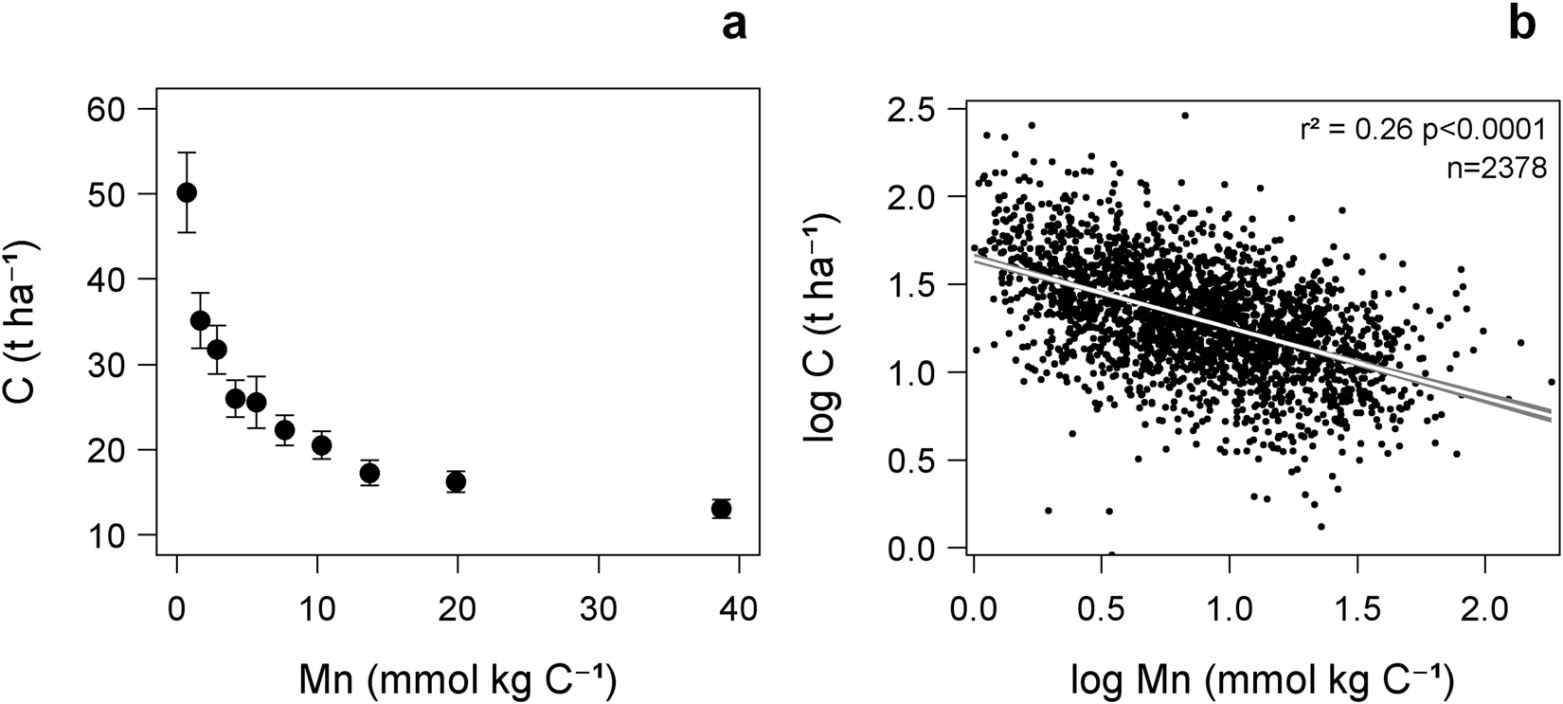
Relationship between Mn and C in humus. Storage of C in humus layers (Sweden), as related to concentrations of exchangeable Mn in the same layers; (**a**) plot of Y-averages for 10 X-percentile classes with Y-error bars representing 95% confidence intervals, and (**b**) log-log regression of C storage vs. Mn in the humus layer for individual plots (n = 2378) including 95% confidence intervals.

**Figure 3:**
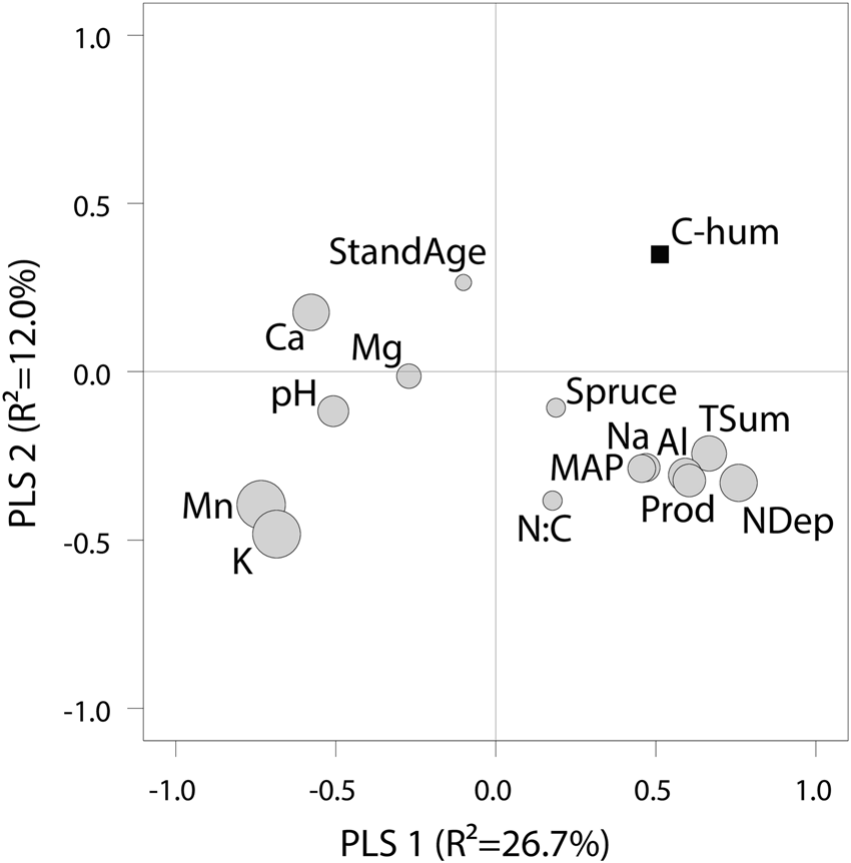
PLS model of C in the humus. Partial least squares (PLS) correlation loadings plot for model explaining C stored in the humus layer. The 1:st PLS axis explains 26.7% and 2:nd PLS axis 12.0% variation and circled areas are proportional to the Variable Importance in Projection. Concentrations of exchangeable nutrients in the humus (mmol kg C^−1^) were log transformed before analysis.

We found that the amount of C stored in the humus layer was primarily and negatively related to exchangeable Mn (normalized to C concentration), whereas 15 other potentially regulating factors including productivity, temperature, and moisture were of less importance (Table 1 and S1). In total, the statistical model explained 44% of the variation in stored C, with 26% attributed solely to exchangeable Mn. In comparison, established ecosystem models failed to predict more than 5% of the variation in below ground C for the same data^3^. The log-linear negative relationship between humus-layer C and exchangeable Mn (Fig. 2a,b) corresponded to a 3-fold difference in stored C across a 50-fold range of observed Mn concentrations. The relationship was consistent after subdividing the data into 9 climate regions (Supplementary Fig. S1) with average temperature sums ranging from 630 to 1480 degree-days (Fig. 1b). Furthermore, C storage at high Mn concentrations (above c. 15 mmol kg C^−1^) appeared to stabilize at an equally low level for all climate regions, indicating little climatic variation in C storage when Mn is available in excess. Accounting for correlation between explanatory variables, we also identified K as an additional strong predictor, however strongly correlated with Mn (Fig. 3, Supplementary Table S2).

**Table 1:** Stepwise GLM (general linear model) of the humus layer C (t ha^−1^) explained by site variables and humus layer chemistry (n = 2378). Concentrations of exchangeable nutrients in the humus (mmol kg C^−1^) were log transformed before analysis.

Significant variables*	Classes	Estimate	SE	t-value	P-value	F-value	Cum. adj-R^2^
Intercept		2.23	0.099	22.6	<0.001	0.0	0.00
Mn		−0.31	0.019	−16.6	<0.001	815.7	0.26
K		−0.49	0.035	−13.9	<0.001	220.7	0.32
Ca		0.29	0.028	10.6	<0.001	151.1	0.36
Spruce (%_BA_)		7.86E-04	1.44E-04	5.5	<0.001	93.3	0.38
Moisture						31.7	0.40
	Dry	−0.20	0.024	−8.4	<0.001		
	Mesic	−0.11	0.013	−8.1	<0.001		
	Moist/Wet	0					
TSum (°days)		1.12E-04	2.42E-05	4.6	<0.001	44.9	0.41
pH		−0.13	0.019	−6.6	<0.001	46.9	0.42
HumusForm						26.2	0.43
	Mor type 1	−0.11	0.018	−6.3	<0.001		
	Mor type 2	−0.04	0.019	−2.3	0.022		
	Moder	0					
Na		0.10	0.023	4.4	<0.001	19.4	0.44
Al		−0.05	0.016	−3.0	0.003	12.9	0.44
MAP (mm)		1.03E-04	3.81E-05	2.7	0.007	6.0	0.44
N:C		−1.80	0.866	−2.1	0.038	4.3	0.44

*All variables are listed in Supplementary Table S1.

A plausible explanation to the Mn effect in the humus layer is the central role of Mn-peroxidase enzymes in fungal-mediated decomposition^26,27^. Manganese-peroxidases are produced by both saprotrophic and mycorrhizal fungi^28^, but only by members of the Agaricomycetes, a class of basidiomycetes that encompasses most mushroom forming fungi^29^. The Mn-peroxidases obtain a high redox potential by reducing H_2_O_2_ and, in turn, oxidize Mn^2+^ to highly reactive Mn^3+^ ions, which diffuse from the fungal hyphae commonly chelated with oxalate^26^. The produced Mn^3+^ ions may oxidize a multitude of different bonds in a variety of organic molecules, leading to the formation of radicals and subsequent depolymerization, or alternatively, condensation of targeted molecules^30^. The Mn-peroxidases constitute the core of the lignin-degrading machinery of basidiomycete wood degraders^19^ and are central in decomposition of melanin^31^ as well as of more unspecified humus compounds in soils^27^, e.g. humic acids^32^. The biological availability of Mn in soils is controlled by weathering of the parent material, loss by leaching, and directed fungal transport to locations of organic matter oxidation^20^. Exchangeable Mn in the humus was only weakly positively (R^2^ = 0.03) related to total Mn in the parent material (Supplementary Fig. S2) suggesting that other factors than mineralogy are critical for Mn availability in the humus. The influence of Mn did not extend into the mineral soil (Supplementary Table S3), indicating that other mechanisms control C storage in the mineral soil than in the humus layer.

Notably, exchangeable K was also negatively related to C (and positively with Mn) in the humus and continued to have a strong relationship with C storage, also in the mineral soil. This poses the question whether limitation of decomposition by K is reasonable. Potassium limitation of decomposition is theoretically possible^33^, with critical concentrations estimated to 20–27 nmol g^−1^ in pine litter^34^, corresponding to exchangeable concentrations of 10–15 nmol g^−1^ ^17^. In the present study exchangeable K ranged from 7–30 nmol g^−1^ (5:th to 95:th percentile), implying that K limitation is possible, but seems unlikely for a majority of the data. Other biogeochemical mechanisms explaining a direct link between potassium and C turnover in the humus are difficult to find. Instead we hypothesize a secondary association between humus K and Mn, either through similar supply mechanisms or correlated leaching patterns.

Our results point towards Mn-dependent oxidation as a potential bottleneck for the turnover of humus layers, with a principal role in the regulation of below ground C sequestration in boreal forests. The importance of other controls of C sequestration, such as plant production, climate, and N-deposition, is subordinate and likely to be primarily indirect, via their effects on Mn-availability and/or fungal activity. Tight coupling of C and N cycling through organic pools implies that the same constraints are likely to regulate N availability and plant productivity^5^. The importance of Mn-dependent oxidation highlights Agaricomycetes as potential keystone organisms in humus decomposition and C storage. Long-term decomposition has been proposed to depend on symbiotically associated ectomycorrhizal fungi^35^ and, consistently, high abundance of ectomycorrhizal Agaricomycetes has been found to correlate negatively with C sequestration in boreal forest humus^7^. The causality of the fungi to Mn relationship needs to be addressed further to elucidate whether Mn-availability controls fungal activity or highly active fungi influence local Mn-availability by active weathering and directed mycelial transport^20^.

## Methods

### Description of the data

The principal data used in this study was obtained from the Swedish Forest Soil Inventory (www.slu.se/markinventeringen) and the National Forest Inventory (www.slu.se/riksskogstaxeringen), which are both carried out recurrently on the same c. 20 000 permanent plots. The inventories represent a random sampling of Swedish land stratified according to 5 geographical regions with different sampling intensity (Fig. 1b). Circular plots (314 m^2^) are organized in quadratic clusters, each encompassing 8 plots (4 in the southwestern region), with increasing side length and distance apart towards the north.

Soil sampling is carried out on a subset of the plots, i.e. humus sampling on c. 10 000 plots and mineral soil sampling on c. 4500 plots. The humus layer is sampled volumetrically using a 10 cm diameter corer throughout the entire depth of O-horizons (to max. 30 cm depth) excluding the litter layer. The sample volume is c. 1.5 litre fresh humus material, which is collected from 1–9 sampling points in a 3.14 m^2^ subplot within each main plot. Mineral soil is collected at fixed depth intervals: 0–10 cm, 10–20 cm, and 55–65 cm. All soil samples are dried to constant weight at 35 °C, homogenized and sieved (<2 mm), and subsequently living and dead roots >1 mm diameter are removed. Chemical analyses are carried out on the fine soil fraction. Chemical variables included in this study were total C and N content (%) analysed using an elemental analyser (LECO CNS-1000 and LECO TruMac CN), the N:C ratio, pH determined in water, exchangeable cations (Ca, Mg, Na, K, and Mn, mmol kg^−1^DM) extracted using 1 M ammonium acetate buffered at pH 7.00, exchangeable Al extracted using 1 M KCl. Both the base cation and Al extracts are analysed by plasma-emission spectrometry (ICP-AES). Exchangeable concentrations were recalculated in relation to C content (mmol kg C^−1^).

Forest data included in the study were site productivity (average wood production, m^3^ ha^−1^ yr^−1^), fraction of Norway spruce (% of basal area, the rest mainly being Scots pine), and stand age (years at 1.3 m height). Other data sources were mean annual precipitation (MAP; from 2012) and total N deposition (NDep; from 1998 representing historical deposition) retrieved from the Swedish Meteorological and Hydrological Institute. Temperature sum (TSum) was defined as the sum of daily mean temperatures exceeding +5 °C and calculated using a function based on latitude and altitude^36^.

In this study, a complete inventory period (2003–2012) was used and selection was made for plots on productive forest land (average production, stem volume over bark >1 m^3^ ha^−1^ yr^−1^), with soil moisture class dry, mesic or mesic/moist (representing an average distance of >2 m, 1–2 m, and 0.67–1 m to ground water, respectively), and terrestrial aerated organic humus forms (mor and moder types; mor 1 comprising >50% OF (Oe) subhorizon, mor 2 comprising 50–75% OF subhorizon and moder comprising <25% OF subhorizon, the rest being OH (Oa) subhorizon), on minerogenic parent material (sediment or till soil). The study material represented c. 66% (15 Mha) of the Swedish productive forest land.

### Estimates of C storage

Storage of organic C in the humus layer was estimated based on the dry weight of the entire humus layer and the C concentration. For the mineral soil, the storage of C in each sampled layer (0–10 cm, 10–20 cm, and 55–65 cm) was estimated using the C concentration (%), bulk density, soil layer thickness, and the volume percentage of stones and boulders:

$${\rm{CarbonStorage}}={\rm{CarbonConc}}\ast \,{\rm{BulkDens}}\ast \,{\rm{LayerThick}}\,\ast \,(100-{\rm{StoneVol}})/100$$

Bulk density (g cm^−3^) for the mineral soil horizons was calculated from the carbon concentration (%) and the depth (cm)^37^:

$${\rm{BulkDens}}=1.5463\,\ast \,\mathrm{EXP}(-0.3130\,\ast \,{{\rm{CarbonConc}}}^{0.5})+0.00207\,\ast \,{\rm{Depth}}$$

The volume of stones and boulders for each plot was determined by the stoniness index measured using a 1 cm diameter probe and subsequently calculating the volume percentage using a transfer function^38^. The storage below 20 cm was estimated by linear interpolation based on the adjacent sampled layers to 50 cm in the mineral soil, or to the maximum soil depth.

### Statistical analyses

Multiple linear explanatory models for C storage were estimated by stepwise GLM using the GLMSELECT procedure in the SAS 9.4 software (Statistical Analysis System Institute, 2002–2012). Variables entered in the models (besides soil moisture and humus form) are listed in Supplementary Table S1. Adjusted R^2^ was used as selection criterion, the significance level for entering or removal of variables was 0.10 based on F-statistics, and the stop criterion was based on the predicted residual sum of squares.

Accounting for multicollinearity, relationships between C storage and environmental variables were investigated by Partial Least Square (PLS) regression models, which extract latent factors in order to maximize the explained variance in the dependent variable. The analysis accounts for covariation among dependent variables and gives a complete picture of all individual relationships. The PLS procedure in SAS 9.4 statistical software (Statistical Analysis System Institute, 2002–2012) was applied using the NIPALS algorithm. All variables were scaled to unit variance in the procedure. The predictive ability of the models were presented as the R^2^, and the X-loadings, correlation loading and Variable Importance in Projection (VIP) were used to evaluate importance of explanatory variables. Two PLS components were used in the model.

Before the statistical analyses, C storages and the concentrations of exchangeable Ca, Mg, Na, K, Mn, and Al were log-transformed (log_10_(*var* + 1)) and individual relationships were checked for linearity.

## Electronic supplementary material

Supplementary information
