# Supplementary material for: Manganese availability is negatively associated with carbon storage in northern coniferous forest humus layers
Source: Sci Rep. 2017 Nov 14;7:15487. doi: 10.1038/s41598-017-15801-y (PMC5686207; doi:10.1038/s41598-017-15801-y)
Supplement: Supplementary file 1 — Supplementary information [file 41598_2017_15801_MOESM1_ESM.pdf]

Supplementary Information for

Manganese availability is negatively associated with carbon storage in northern coniferous forest humus layers

J. Stendahl, B. Berg, B. D. Lindahl

Corresponding author: johan.stendahl@slu.se

Contents:

Figures S1 and S2
Tables S1 - S3

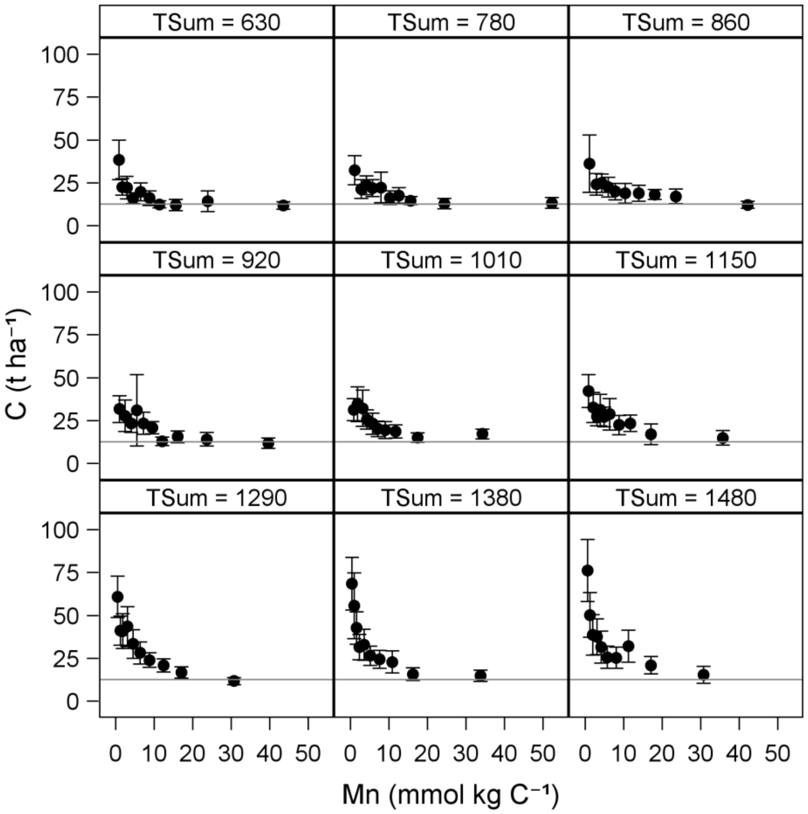

Figure S1. Storage of C in the humus layer (Sweden), as related to concentrations of exchangeable Mn in the same layer for different climate regions based on temperature sum.

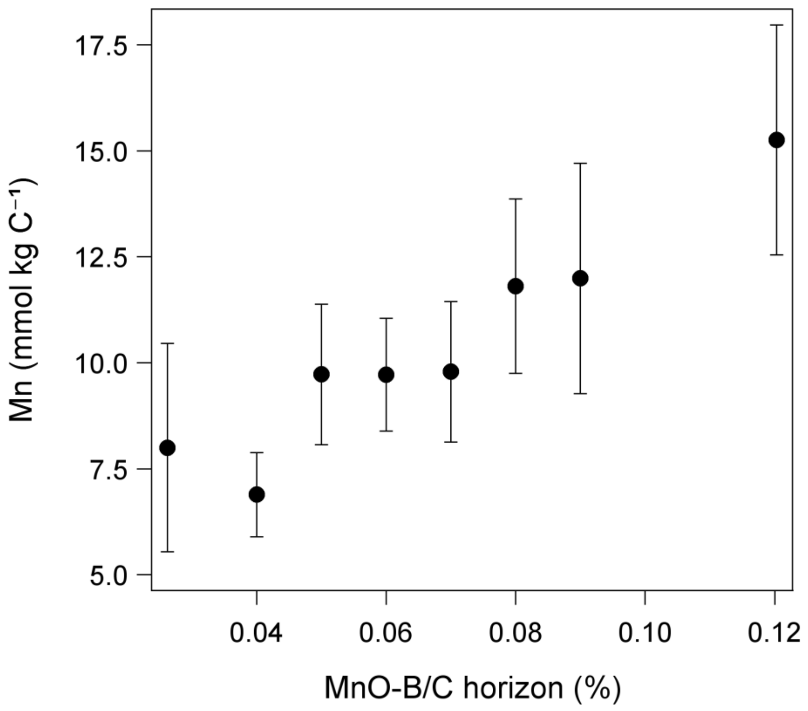

Fig. S2. Concentration of exchangeable Mn in the humus layer versus concentration of total Mn (MnO) in the parent material (transition between B and C horizon).

Table S1. Descriptive statistics of data from the Swedish Forest Soil Inventory. Concentrations of exchangeable nutrients are given per unit C (mmol kg C^{-1}) in different soil layers (*hum* denotes the humus layer, while *10*, *20* and *65* denote the 0-10, 10-20 and 55-65 cm layers in the mineral soil). Variables used as input in the statistical models of C storage in the humus and soil by GLM and PLS are indicated in separate columns.

Variable	n	Mean	SD	Skew	Q1	Q3	Humus	Soil
C-hum (t ha^{-1})	2378	25.8	22.5	3.6	12.5	31.8	Y	
C-$soil_{50cm}$ (t ha^{-1})	2206	42.4	30.3	3.1	23.4	53.1		Y
TSum (°days)	2378	1060	273	0.1	843	1320	X	X
MAP (mm)	2378	884	139	1.1	792	950	X	X
NDep (kg ha^{-1})	2378	5.3	3.1	1.1	2.9	6.8	X	X
Prod (m^3 ha^{-1} yr^{-1})	2378	5.6	2.8	0.8	3.4	7.3	X	X
Spruce ($\%_{BA}$)	2378	37.9	36.4	0.5	0.0	70.0	X	X
StandAge (yr)	2378	62.6	47.7	0.9	25.0	94.0	X	X
pH-hum	2378	3.8	0.4	1.5	3.6	4.0	X	X
N:C-hum	2378	0.033	0.008	0.9	0.028	0.038	X	X
Ca-hum	2378	170	270	17.4	86.6	187	X	X
Mg-hum	2378	43.4	38.6	11.4	27.7	49.3	X	X
K-hum	2378	50.1	26.8	6.7	35.6	59.6	X	X
Na-hum	2378	7.8	7.1	4.6	3.8	9.2	X	X
Mn-hum	2378	10.5	12.4	3.8	2.8	13.5	X	X
Al-hum	2378	29.3	30.8	2.6	10.0	35.4	X	X
pH-10	2200	4.4	0.4	1.2	4.2	4.6		X
N:C-10	2200	0.050	0.017	1.7	0.040	0.057		X
Ca-10	2200	147	361	14.0	25.0	138		X
Mg-10	2200	51.9	382	44.6	15.0	44.6		X
K-10	2200	34.7	38.1	25.7	20.0	42.2		X
Na-10	2200	22.1	30.4	23.4	10.5	26.3		X
Mn-10	2200	6.7	18.0	7.8	0.5	5.5		X
Al-10	2200	450	313	2.5	267	535		X
pH-20	2058	4.7	0.4	1.2	4.5	4.9		X
N:C-20	2058	0.055	0.020	2.2	0.043	0.063		X
Ca-20	2058	188	665	13.6	20.0	127		X
Mg-20	2058	52.6	187	13.0	9.6	36.2		X
K-20	2058	34.6	34.4	6.0	17.6	39.7		X
Na-20	2058	25.5	30.0	7.5	11.0	30.7		X
Mn-20	2058	5.3	13.0	7.3	0.3	4.5		X
Al-20	2058	407	330	3.6	211	508		X
pH-65	1494	5.0	0.4	2.0	4.8	5.2		X
N:C-65	1494	0.097	0.081	4.4	0.054	0.113		X
Ca-65	1494	878	2937	6.0	49.4	302		X
Mg-65	1494	403	2121	9.9	13.6	70		X
K-65	1494	129	228	6.3	33.6	135		X
Na-65	1494	100	206	7.8	23.9	98		X
Mn-65	1494	7.8	17.9	7.4	0.7	7.6		X
Al-65	1494	524	511	8.4	274	628		X
MnO-B/C (%)	1100	0.064	0.028	1.4	0.050	0.080		

Table S2. Loadings and Variable Importance in Projection (VIP) for PLS model of C stored in the humus layer explained by site variables and chemistry in the humus layer.

	X-loadings		
Variable	PLS 1	PLS 2	VIP
Mn	-0.37	-0.36	1.82
K	-0.35	-0.44	1.77
NDep	0.38	-0.30	1.13
Ca	-0.29	0.16	1.09
TSum	0.34	-0.22	1.00
Al	0.30	-0.28	0.95
Prod	0.31	-0.30	0.90
pH	-0.26	-0.11	0.82
Na	0.24	-0.26	0.70
MAP	0.23	-0.26	0.69
Mg	-0.14	-0.01	0.56
N:C	0.09	-0.35	0.40
Spruce ($\%_{BA}$)	0.10	-0.10	0.39
StandAge	-0.05	0.24	0.32

Table S3. Stepwise GLM analysis of C stored in the mineral soil to 50 cm depth explained by site variables and chemistry in different soil layers (*hum* denotes the humus layer, while *10*, *20* and *65* denote the 0-10, 10-20 and 55-65 cm layers in the mineral soil). Concentrations of exchangeable nutrients (mmol kg C^{-1}) were log transformed before the analysis.

Significant variables*	Estimate	SE	t-value	P-value	F-value	Cum. adj-R^2
Intercept	2.53	0.095	26.5	<0.001	0.0	0.00
K-20	-0.25	0.026	-9.5	<0.001	443.6	0.23
Mg-hum	0.10	0.033	3.1	0.002	127.9	0.29
Na-10	-0.11	0.026	-4.1	<0.001	72.6	0.32
Na-hum	0.16	0.027	6.0	<0.001	87.3	0.36
Al-65	-0.08	0.012	-6.7	<0.001	40.1	0.38
pH-10	-0.14	0.019	-7.8	<0.001	36.3	0.39
pH-hum	0.09	0.018	5.1	<0.001	43.7	0.41
Mg-65	0.06	0.009	7.0	<0.001	26.1	0.42
Ca-20	-0.03	0.016	-2.2	0.031	38.1	0.43
N:C-hum	3.64	0.781	4.7	<0.001	17.1	0.44
N:C-20	-1.00	0.280	-3.6	<0.001	19.8	0.45
Al-20	-0.06	0.015	-4.2	<0.001	15.2	0.45
Spruce ($\%_{BA}$)	0.05	0.014	3.8	<0.001	12.1	0.45
Na-20	-0.11	0.025	-4.2	<0.001	11.3	0.46
Mg-10	-0.08	0.024	-3.3	0.001	11.1	0.46

*All entered variables are found in Table S1.
